# Supplementary material for: Emergent normal fluid in the superconducting ground state of overdoped cuprates
Source: Nat Commun. 2024 Jun 10;15:4939. doi: 10.1038/s41467-024-49325-7 (PMC11164957; doi:10.1038/s41467-024-49325-7)
Supplement: Supplementary file 1 — Supplementary Information [file 41467_2024_49325_MOESM1_ESM.pdf]

This PDF file includes:

Supplementary Text

SI I. Estimation of the doping level

SI II. The normalization of dI/dV spectra in different samples

SI III. The increase of zero-bias DOS with the overdoping process

SI IV. The dataset analyzed in the main text

SI V. Extraction of the zero-bias QPI intensity near antinode in different samples

SI VI. The bias dependence of QPI

SI VII. The arc-like QPI pattern in the finite energy of UD-20K and OP-32K samples

SI VIII. Normal carrier QPI in separated areas with different local pseudogap sizes

SI IX. Estimating the percentage of normal gapless region in the OD-15K sample

SI X. The mean-field simulation of a disordered d-wave superconductor

SI XI. Variable temperature experiment with atomic scale tracking

SI XII. Comparison between the QPI intensity and simulated spectral function

SI XIII. The evaluation of the superconducting gap size

SI XIV. Absence of zero-energy arc-like QPI in bilayer cuprate Bi-2212

SI XV. Un-symmetrized QPI dataset

SI XVI. The QPI pattern with different bias modulations

Figs. S1 to S15

Fig. S1| Characterization of the Bi-2201 samples

Fig. S2| Averaged spectra normalized by the mean DOS at different energies

Fig. S3| The relation between the pseudogap size and zero-bias DOS

Fig. S4| The dataset analyzed in the main text

Fig. S5| Extraction of zero-bias QPI intensity

Fig. S6| Bias dependence of the QPI

Fig. S7| QPI pattern of UD-20K and OP-32K samples

Fig. S8| Normal carrier QPI in different areas

Fig. S9| Spatial distribution of the QPI intensity in the superconducting and normal states

Fig. S10| Estimating the percentage of normal region in the OD-15K sample

Fig. S11| Variable temperature experiment with atomic scale tracking

Fig. S12| Comparison between experimental and simulated results

Fig. S13| Distribution of superconducting gap and quasiparticle broadening in OD-15K and OP-32K samples

Fig. S14| QPI pattern in an overdoped Bi-2212

Fig. S15| The un-symmetrized and symmetrized QPI patterns at zero bias

Fig. S16| Arc-like QPI pattern at different bias modulations on the OD-28K sample

Tabs. S1 to S2

Tab. S1| The details of La and Pb content in every Bi-2201 samples

Tab. S2| The parameters of spectral grid supporting the analysis in the main text

References

Supplementary Text

I. Estimation of the doping level

To evaluate the doping level of the Bi-2201 samples, we determine their T_c by using temperature dependent susceptibility curves displayed in Fig. S1b. Figure S1c displays a non-superconducting transport behavior in the OD-NSC sample, consistent with Ref. [1]. An empirical relation between the hole density and T_c has been established[2,3], in which a bell-shaped parabolic $p - T_c$ relation is expressed as $\frac{T_c}{T_c^{\max}} = 1 - \frac{(p-0.16)^2}{(0.10-0.16)^2}$. The hole densities of p = 0.13, 0.16, 0.18, 0.19, and 0.21 are estimated by using this empirical formula. The doping level of the non-superconducting sample is estimated by the same method described in Ref. [4]. The temperature dependent resistance of OD-NSC sample follow the scaling law $\rho_{ab}(T) = a_1 T^{4/3} + a_2$, where the red dashed line indicates the fitting result of $a_1 = 3.7 \times 10^{-4} \mathrm{m\Omega \cdot cm \cdot K^{-\frac{4}{3}}}, a_2 = 0.33\ \mathrm{m\Omega \cdot cm}$. It is consistent with the behavior in extremely overdoped Bi-2201, where the two-dimensional ferromagnetic fluctuation might be responsible for the scaling factor of $4/3$ (Ref. [1]).

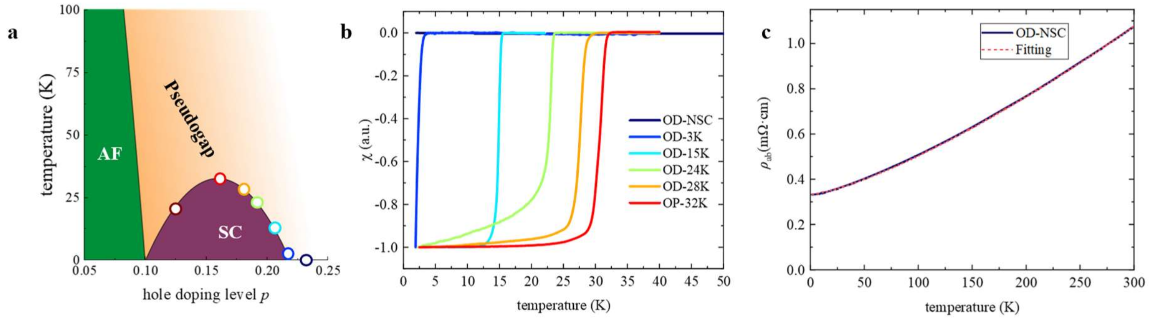

Fig. S1| Characterization of the Bi-2201 samples. a, The phase diagram of Bi-2201 with all samples marked by the corresponding circles. **b,** The temperature dependent susceptibility of all samples measured by SQUID. The susceptibilities are normalized to unity. **c,** The resistivity versus temperature curve of the

OD-NSC sample showing a metallic behavior down to 1.2 K. The red dashed line displays the fitting result of $\rho_{\mathrm{ab}}(T) \propto a_1 T^{4/3} + a_2$.

II. The normalization of dI/dV spectra in different samples

The data in Fig. 1b encompass a diverse range of samples, from OP-32K to OD-NSC, each displaying distinct spectral features. To elucidate the evolution of zero-bias DOS from a deep gap to a peak-like vHS, we normalized the spectra by the high-energy background that is not strongly affected by the pertinent low-energy physics. Figure S2 displays the averaged spectra normalized at ±60 mV, ±70 mV and ±80 mV. The general trend of the spectral evolution is not sensitive to the choice of normalization parameter as long as the energy is large enough.

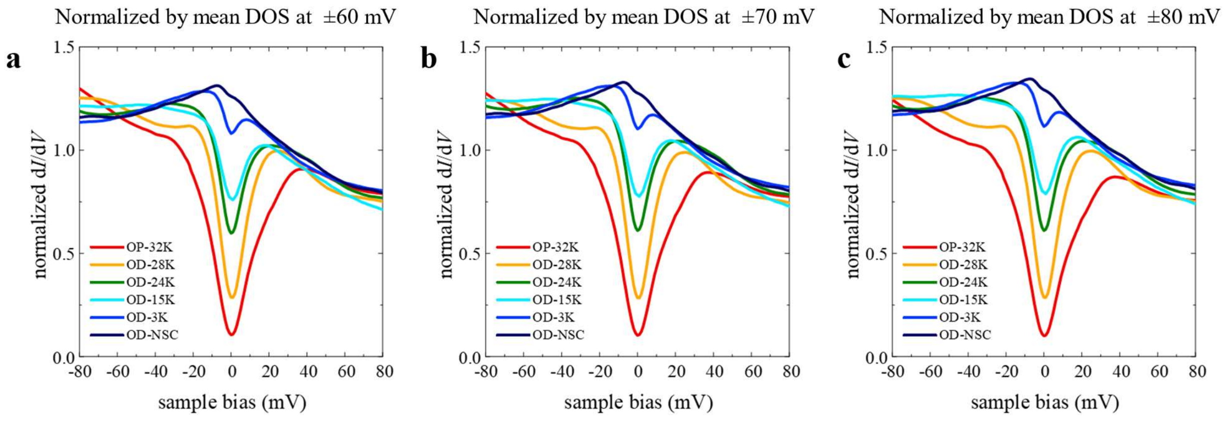

Fig. S2| Averaged spectra normalized by the mean DOS at different energies. a-c, The averaged spectra normalized by the mean DOS at ±60 mV, ±70 mV and ±80 mV, respectively. The trends are insensitive to the normalization parameter.

III. The increase of zero-bias DOS with the overdoping process

To further investigate the evolution of the zero-bias DOS across different samples, we display the relation between zero-bias DOS and pseudogap size in from UD-20K to OD-15K samples. The

zero-bias DOS is nearly unchanged with the pseudogap size in the underdoped and optimal doped samples. When the system enters the overdoped regime, the zero-bias DOS increases with decreasing local pseudogap gap size remarkably, as shown in Fig. S3. The spectra with similar pseudogap size in different samples also exhibit different zero-bias DOS, implying the impact of average doping levels. A branch of gapless electronic sector exists in the whole sample instead of localized in some rare region with higher doping level. Such a trend is consistent with the physical picture that the emergent normal fluid becomes more significant with overdoping.

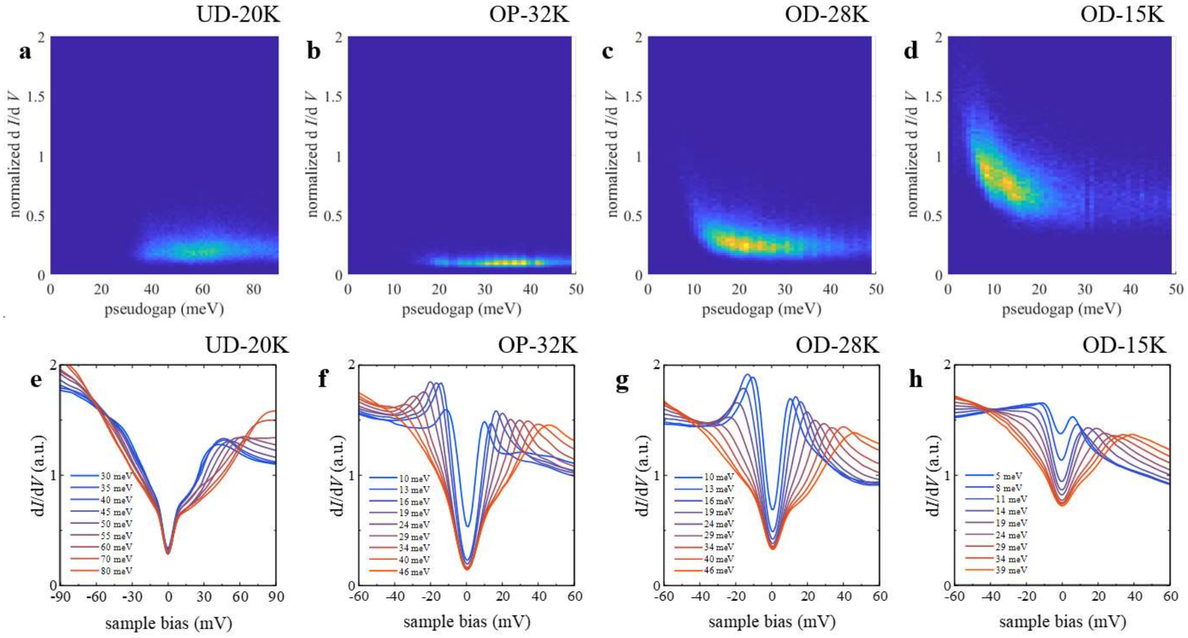

Fig. S3| The relation between the pseudogap size and zero-bias DOS. a-d, The heatmap of the zero-bias DOS and the pseudogap size from UD-20K to OD-15K samples. **e-h,** The averaged spectra sorted by the pseudogap size in the corresponding sample in **a-d**.

IV. <u>The dataset analyzed in the main text</u>

Figures S4h-n display the zero-bias conductance map $g(\mathbf{r}, 0\ \mathrm{mV})$ analyzed in the main text. The high-quality spectroscopic mapping data ensures satisfactory signal-to-noise ratio for the QPI

images. The topographies taken simultaneously with the spectroscopic mapping are displayed in Figs. S4a-g The atomically resolved topographic image demonstrates the excellent condition of the STM tip. As shown in Fig. S4h-n, the long-range checkerboard order gradually diminishes into short-range glassy patterns with isolated plaquette in the overdoping process. The real-space DOS map in the overdoped side displays a highly spatially inhomogeneity and the absence of the long-range periodicity. The highly disordered patterns in the overdoped sample are also consistent with the picture of the disorder induced pair-breaking scattering.

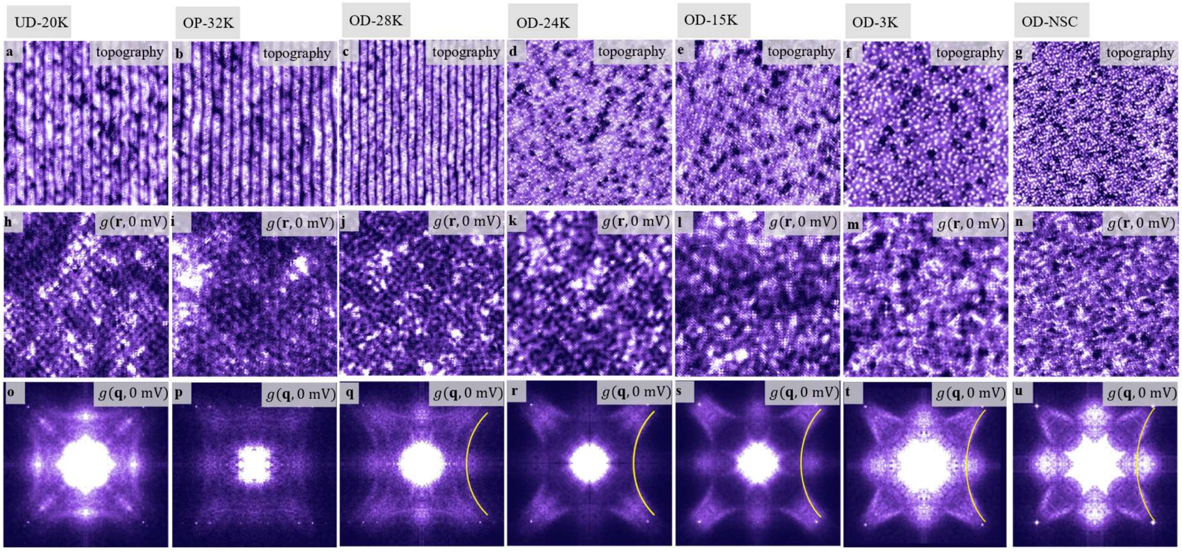

Fig. S4| The dataset analyzed in the main text. a-g, Topography of UD-20K, OP-32K, OD-28K, OD-24K, OD-15K, OD-3K and OD-NSC samples, respectively. **h-n**, Zero-bias conductance maps of these samples. **o-u**, The Fourier transform of **h-n**.

V. Extraction of the zero-bias QPI intensity near antinode in different samples

To address the doping evolution of nearly-circular QPI intensity, which is the weight of normal fluid, we extracted the QPI intensity near antinode by the definition below:

$$I_{\mathrm{QPI}-} = \int_0^{\frac{\pi}{9}} g(k = k_{\mathrm{F}}, \theta_k, E = 0\ \mathrm{mV})\mathrm{d}\theta_k$$

The definition of θ_k and the boundary of $\frac{\pi}{9}$ are labeled in Fig. S5a which can capture the major weight of the normal carrier QPI near the antinodes. The conductance maps across various samples are normalized by the conductance at ± 70 mV, being the same as the method described in the caption of Fig. 1b

Figure S5b displays the systematic accumulation of normal carrier QPI intensity with increasing doping. This is consistent with the picture of emergent normal fluid induced by the disordered pair-breaking scattering. When the quasiparticle scattering rate $\hbar/\tau$ exceeds the energy gap Δ, the generated DOS at Fermi energy is $N_S(E_F) = N_0\sqrt{1-(\tau\Delta/\hbar)^2}$, where N_0 is the DOS of the normal band at Fermi energy. With the increasing of pair breaking rate in the overdoping process, the normal fluid component emerging from the disorder scattering is also enhanced.

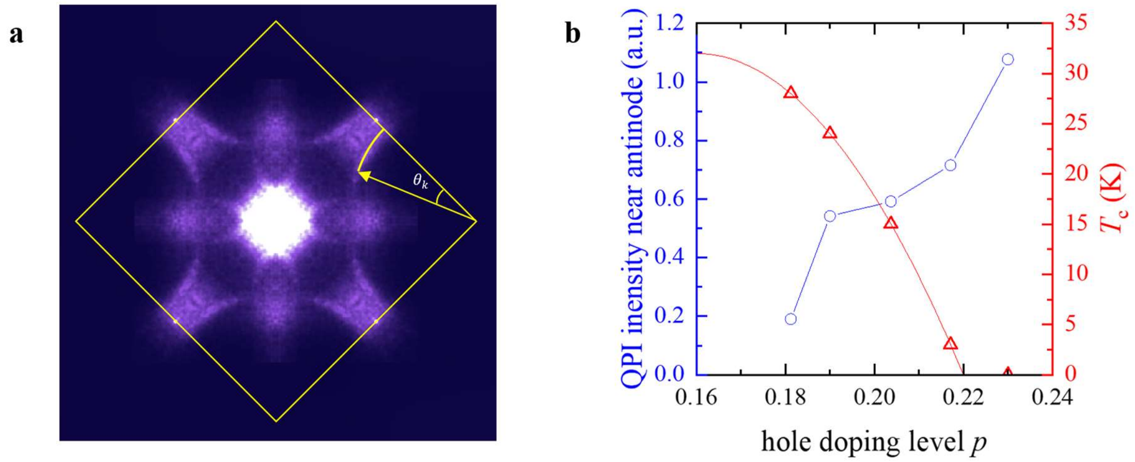

Fig. S5| Extraction of zero-bias QPI intensity. a, The zero-bias QPI of normal carrier g(q, E = 0 mV), with definition of θ_k labeled. **b**, The extracted normal carrier QPI intensity near the antinode as a function of doping level, together with the T_c value of each sample.

VI. The bias dependence of QPI

To reveal the bias dependence of QPI, in Fig. S6a-d we plot the cross-sectional $g(q_y, E)$ on four overdoped samples with q_x fixed at $\frac{2}{3}Q_{\mathrm{Bragg}}$, as illustrated by the cyan surface in Fig. 3c in the main text. The intensity is normalized by the spatially averaged conductance at the corresponding bias. All the maps show electron-type parabolic dispersions near the vHS at the M point, and they systematically shift up with increasing hole density, as summarized in Fig. S6e. The energy and doping dependences of the QPI features are also highly consistent with the normal carrier picture.

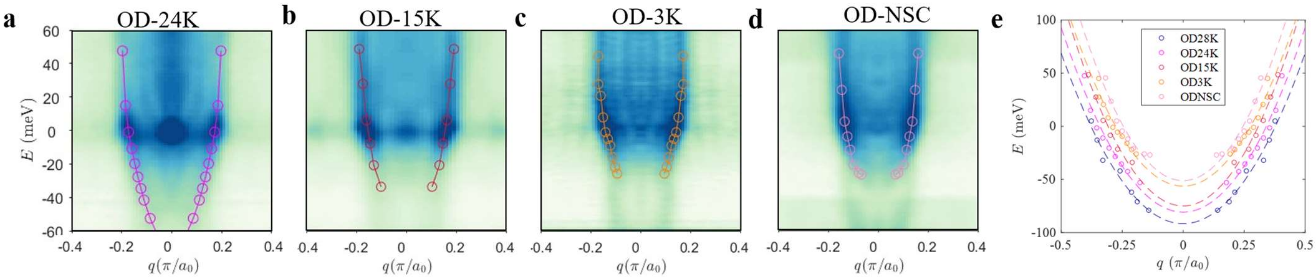

Fig. S6| Bias dependence of the QPI. a-d, The intensity of QPI on the cross-section near the antinodal region as indicated by the cyan surface in Fig. 4g in the main text. The van Hove singularity is cut into an electron-type parabolic dispersion. The three overdoped samples all show parabolic dispersion with a systematic shift of E_F. **e,** The extracted *E*-*q* relations from **a-d** with parabolic dashed lines confirming the electron-type dispersion and a rigid band shift.

VII. The arc-like QPI pattern in the finite energy of UD-20K and OP-32K samples

Figure S7 displays more complete energy-dependent QPI results on the underdoped and optimally doped samples. The arc-shaped patterns are weak but evident at high energies in the UD-20K and OP-32K samples. The QPI at negative biases of the two samples are similar with that

at the E_F of overdoped samples, considering the shift of E_F with increasing hole doping. These comparisons indeed provide a more comprehensive understanding of the QPI phenomena in cuprate. The arc-like QPI patterns at finite biases in underdoped samples indicate that they derive from the normal carrier band structure and are not exclusive to the overdoped one. However, the finding that arc-shaped QPI exists at E_F of the overdoped sample indicate the breaking of Cooper pairs and the recovery of normal carrier band even in the superconducting ground state.

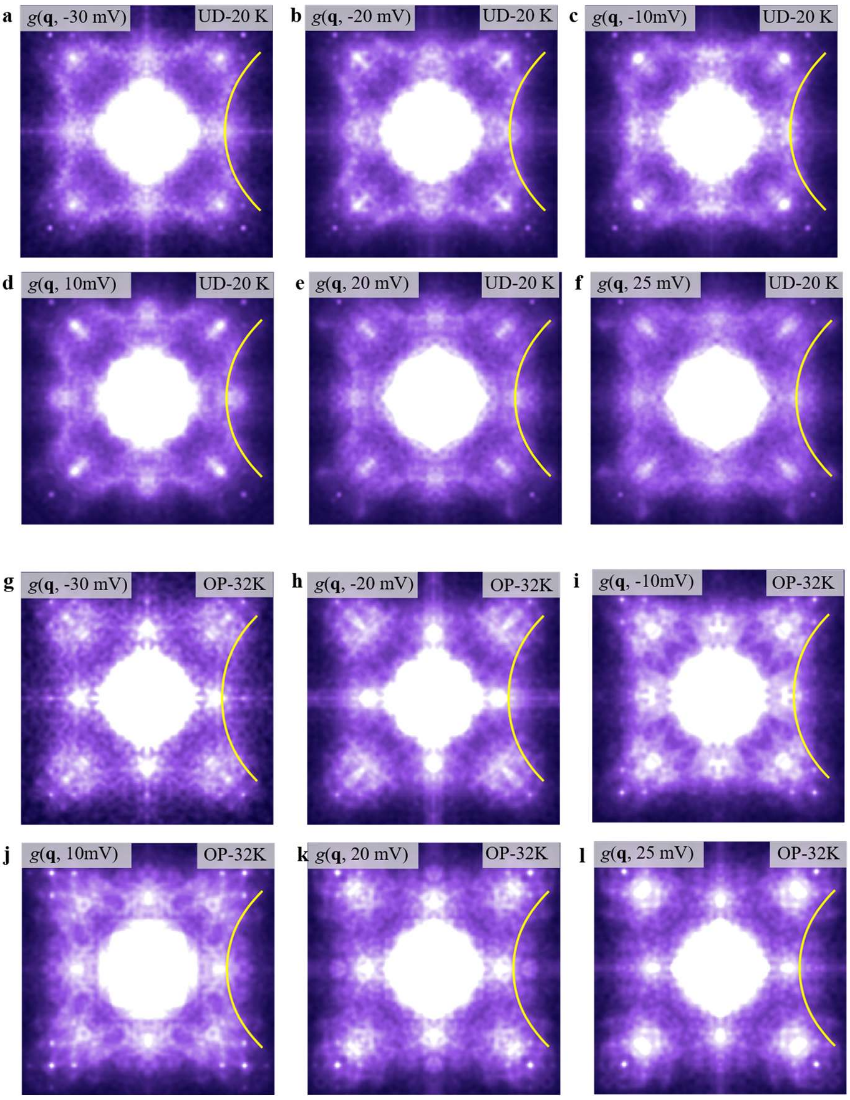

Fig. S7| QPI patterns of the UD-20K and OP-32K samples. a-f, The FT of conductance maps at -30, -20, -10, 10, 20 and 25 mV in UD-20K sample. **g-l**, The FT of conductance maps at -30, -20, -10, 10, 20 and 25 mV in OP-32K sample.

VIII. Normal carrier QPI in separated areas with different local pseudogap sizes

To analyze the spatial inhomogeneity of the normal carrier QPI, we bin the data of the OD-15K sample in eight groups with averaged pseudogap ranging from 6 meV to 29 meV. Then we carry out the FT analysis on the $g(\mathbf{r}, 0 \text{ mV})$ in each area, as shown in Figs. S8b-i, The QPI intensities near antinodes in different areas are extracted by the similar method described in Sec. II. The weight of normal carrier QPI decreases with increasing pseudogap size, which is consistent with the pair-breaking scattering picture. The scattering between the antinodes with sign-reversing order parameter can contribute to the pair breaking scattering, and its strength is enhanced in the overdoping process because the approaching of E_F to the vHS leads to larger DOS. Nevertheless, the normal carrier QPI still exists in the largest pseudogap area, as shown in Fig. S8i, indicating that normal carriers exist in all the areas. It is also verified by the pseudogap clustering of averaged dI/dV curves in Fig. 1e-h. The increase of zero-bias DOS with overdoping is not merely due to the decrease of pseudogap size, but also originates from the emergence of quantum normal fluid.

To explore the temperature dependence of the emergent normal fluid, we extract and compare the normal carrier QPI intensity at $T = 5$ K and 23 K in OD-15K sample. As shown in Fig. S9, the normal carrier QPI distribution has the same trend below and above T_c. The temperature independence reinforces our conclusion that the emergent normal fluid is generated by the disorder scattering.

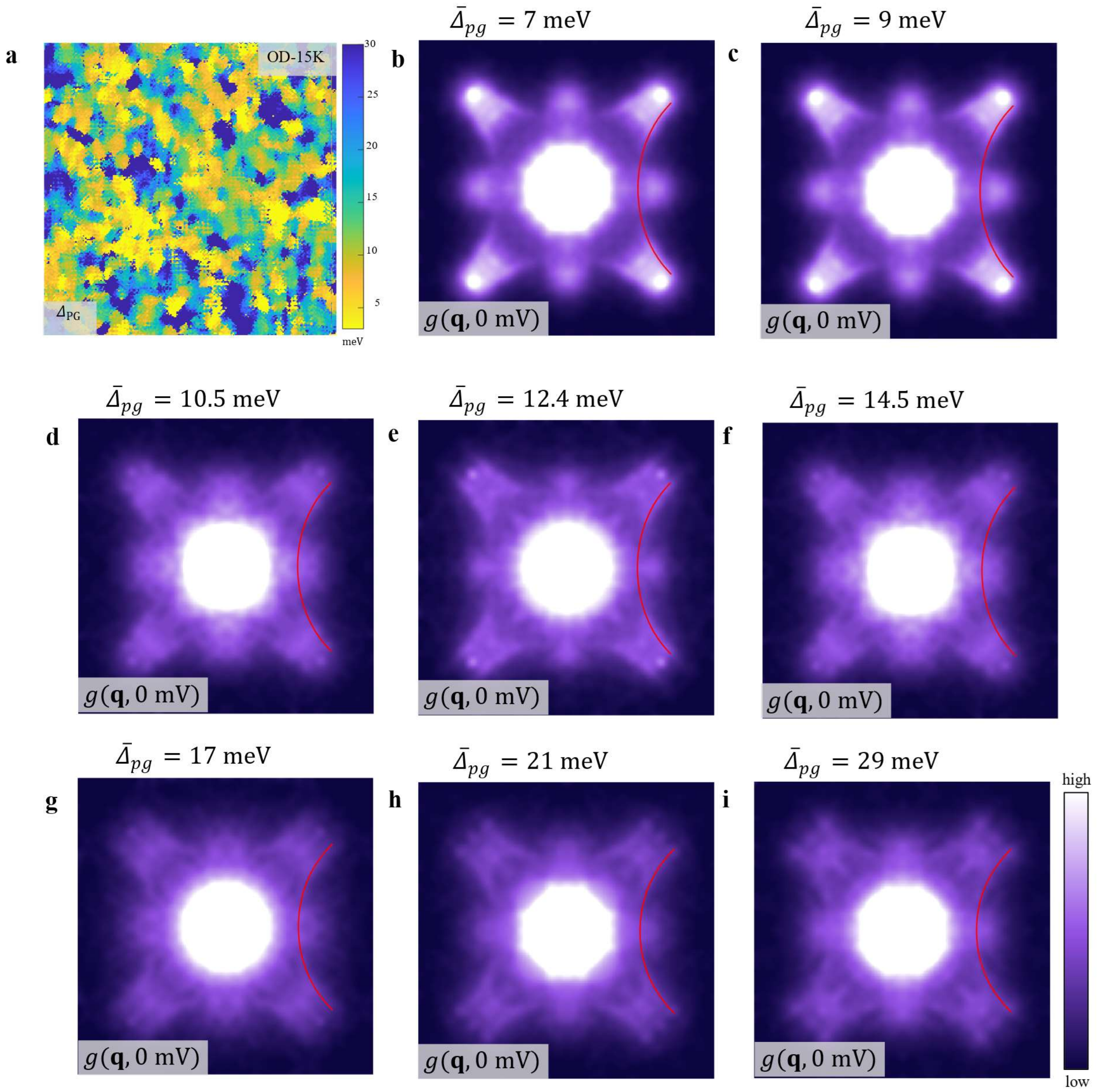

Fig. S8| Normal carrier QPI in different areas. a, The distribution of local pseudogap in the OD-15K sample. **b-i**, the zero-bias QPI pattern in different pseudogap size clustering.

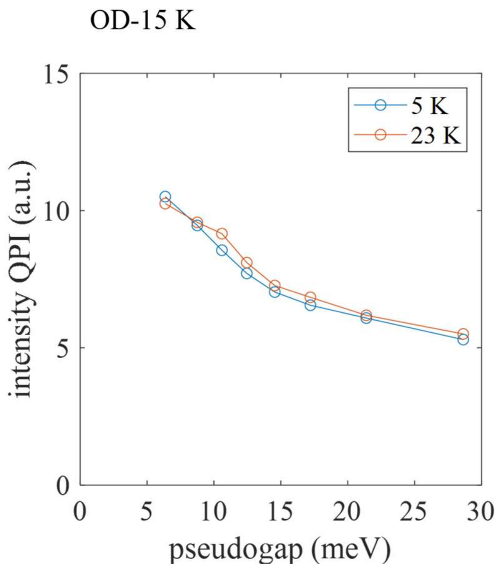

Fig. S9| Spatial distribution of QPI intensity in the superconducting and normal states. The evolution of zero-bias QPI intensity near antinodes with the averaged local pseudogap size at $T = 5$ K and 23 K in OD-15K sample.

IX. Estimating the percentage of normal gapless region in the OD-15K sample

In the overdoped non-superconducting limit, the spectra shows vHS peak near E_F[4]. The low energy DOS suppression in the superconducting region will be filled up by thermal activations. Therefore, we compare the low energy DOS (between ± 6 mV) at the same location for below and above T_c. To ensure the point-to-point tracking accuracy, we employ the Lawler-Fujita algorithm[5]. We mark all gapless vHS-peak-type spectra in Fig. S10b by white color, which occupy about 10% of the whole field of view. The local pseudogap map is displayed in Fig. S10c. The vHS-peak-type spectra are mainly located at the region with smaller pseudogap, indicating a higher local doping level.

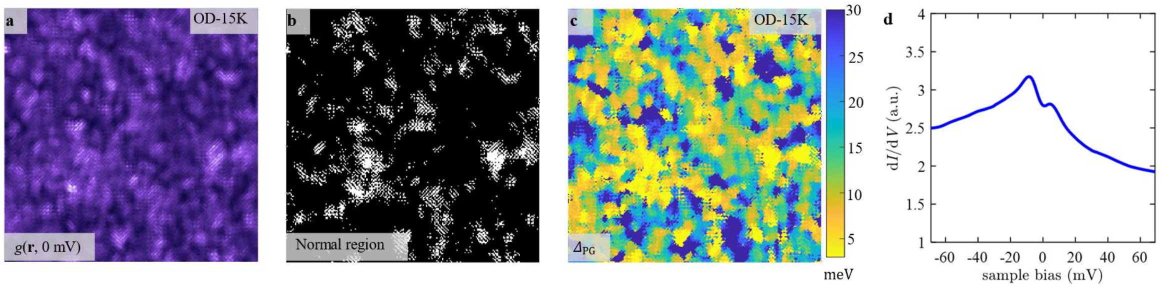

Fig. S10| Estimating the percentage of normal region in the OD-15K sample. a, The zero-bias conductance map of the OD-15K sample measured at $T = 23$ K above T_c. **b,** The gapless region is marked by white color, which occupies about 10% of the whole area. The field of view is the same as **a**. **c,** The local pseudogap size map of the field of view in **a**. **d**, The averaged spectrum of gapless area.

X. The mean-field simulation of a disordered *d*-wave superconductor.

To evaluate the role of non-magnetic disorder on a *d*-wave superconductor, we consider a model including electron hopping, disorder potential and effective interaction terms. The Hamiltonian is:

$$H = -\sum_{i,j,\sigma} t_{ij}\left(c_{i\sigma}^{\dagger} c_{j\sigma} + h.c.\right) + \sum_{i,\sigma} (w_i - \mu) c_{i\sigma}^{\dagger} c_{i\sigma} + J \sum_{\langle ij \rangle} \left(\mathbf{S}_i \cdot \mathbf{S}_j - \frac{1}{4} n_i n_j\right),$$

where t_{ij} is the hopping integral between sites i and j on a square lattice. To reproduce the Fermi surface in hole-doped cuprate, we take $t_{ij} = 1$ for nearest-neighbor sites, $t_{ij} = -0.35$ for next-nearest-neighbor sites and $t_{ij} = 0$ for all further neighbors. J represents the amplitude of interaction yielding *d*-wave superconducting pairing, and we fix $J = 0.8$ in our simulation. The random potentials w_i mimic the effects of disorder induced by point impurities: on a randomly chosen fraction n_{imp} sites $w_i = w > 0$, denoting the sites in the presence of impurity; on other sites $w_i = 0$. We fix $w = 1$ throughout our calculation. In the simulation of overdoped cuprate, we choose $n_{imp} = 0.25$ to sufficiently incorporate the effect of disorder. As a contrast, we use a

smaller fraction of impurity sites $n_{imp} = 0.0625$ in the simulation of underdoped cuprate. The comparison of the results in two cases stresses the crucial role of disorder scattering in reproducing the Fermi surface observed in QPI measurements.

To solve the ground state of the Hamiltonian, we employ a self-consistent mean-field approximation to decouple the interaction terms into the singlet superconducting channel. After the mean-field decoupling, the interaction terms read:

$$H_{d-sc} = -\frac{1}{2}\sum_{\langle ij\rangle} \Delta_{ij}\left(c_{i\uparrow}^{\dagger}c_{j\downarrow}^{\dagger} - c_{i\downarrow}^{\dagger}c_{j\uparrow}^{\dagger}\right) + h.c.,$$

where $\Delta_{ij}= J\langle c_{j\downarrow}c_{i\uparrow} - c_{j\uparrow}c_{i\downarrow}\rangle$ is the *d*-wave superconducting pairing amplitude that has been calculated self-consistently. We perform the mean-field calculation numerically in a finite system of size 40×40 with periodic boundary condition. The result is the average over 64 distinct disorder configurations. We have checked that the number of disorder configurations is sufficiently large to produce the converged results of physical observables upon further increasing disorder configuration number.

XI. Variable temperature experiment with atomic scale tracking

To quantitatively explore the correlation between the long-range superconducting order and normal carrier QPI, we carry out the QPI experiment on the identical areas with the same setup parameters at $T = 5$ K and 23 K. We first heat up the STM head to 23 K and collect the high-quality QPI data after the thermal drift is relaxed. Then the STM is cooled down to the base temperature $T = 5$ K and the same experiment is carried out on the same area. The condition of tip apex is unchanged in the two data sets, which guarantees the same tunneling matrix and make the quantitative comparison reliable. Besides, the Lawler-Fujita algorithm is applied on the topography simultaneously acquired with the dense spectral grid. The topographic data and $g(\mathbf{r}$, 0

mV) of the OD-15K and UD-20K samples at 5 K and 23 K are displayed in Fig. S11, respectively, showing atom-to-atom precision.

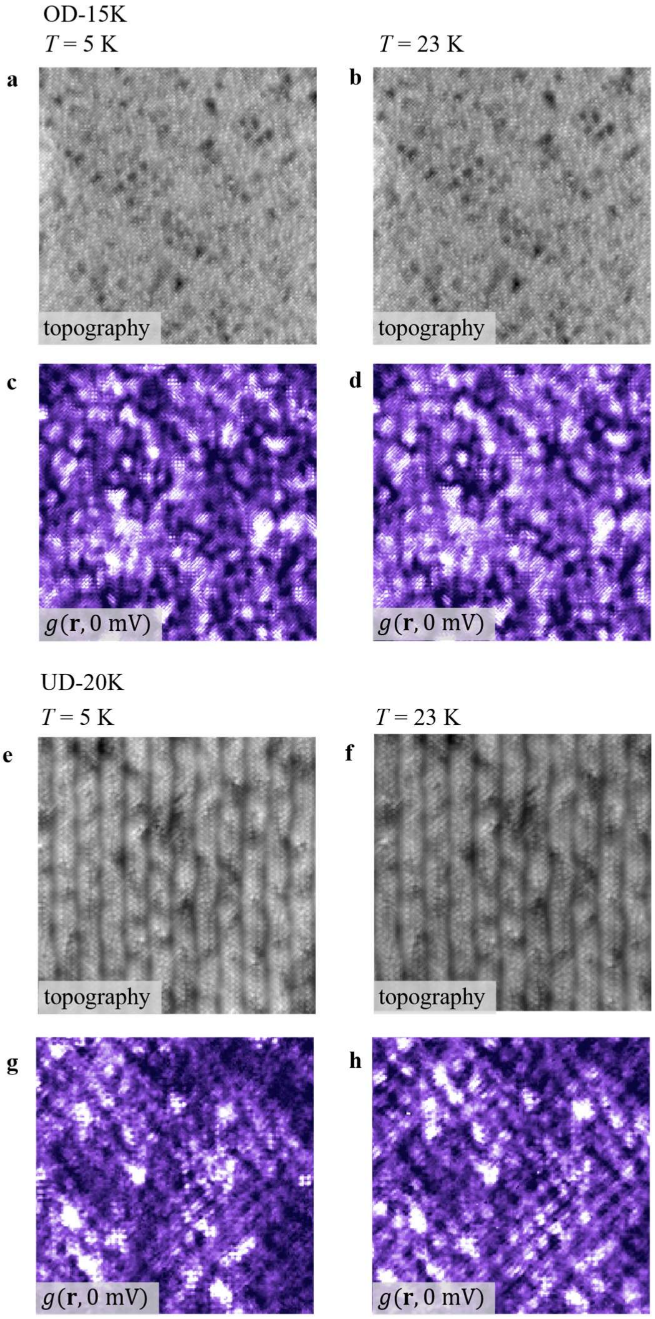

Fig. S11| Variable temperature experiment with atomic scale tracking. a-b, The topographic image on the OD-15K sample at $T = 5$ K and 23 K. **c-d**, The g(r, 0 mV) on the OD-15K sample at $T = 5$ K and 23 K. **e-h**, Same data set with **a-d** on the UD-20K sample.

XII. Comparison between the QPI intensity and simulated spectral function

The simulated spectral function $A(\mathbf{k},\omega)$ is obtained by the summation of the Fourier components of all eigenstates. The spectral functions at E_F, $A(\mathbf{k},\omega=0)$, of the UD/OD configurations with/without superconducting order are shown in Fig. 7a-d. The angle dependences of $A(\mathbf{k}=\mathbf{k}_f,\omega=0)$ are extracted and displayed in Figs. S12a and S12c.

In the underdoped superconducting state, the excitation is dominated by the nodal quasiparticles, which has been proposed by previous theoretical consideration[6]. When we remove the superconducting order, the Fermi surface recovers, as shown in Fig. 4c and the red line in Fig. S12a. The spot-like feature of spectral function in the underdoped case originates from the finite-size effect. The QPI intensity also reveals an unchanged nodal spectral weight and enhanced spectral weight outside nodal region with temperature up to $T = 23$ K > T_c. It is consistent with the recovering of full Fermi surface in the simulation after removing the superconducting order.

In the overdoped configuration, which has strong disorder scattering, the full Fermi surface is present, both in the superconducting and normal states, as shown in Fig. S12c. It indicates that the full Fermi surface has been established in the superconducting ground state because of the temperature-independent pair-breaking scattering. The QPI intensity of the OD-15K sample also reveals a temperature-independent behavior with relatively high spectral weight in the antinodal region, where the peak near $\theta_k = 45°$ is caused by the cross-vHS (π,π) scattering[4,7]. As shown in Fig. S12d, the temperature-independence of antinodal QPI at zero bias supports that disorder scattering is the mechanism for the generation of antinodal normal carrier.

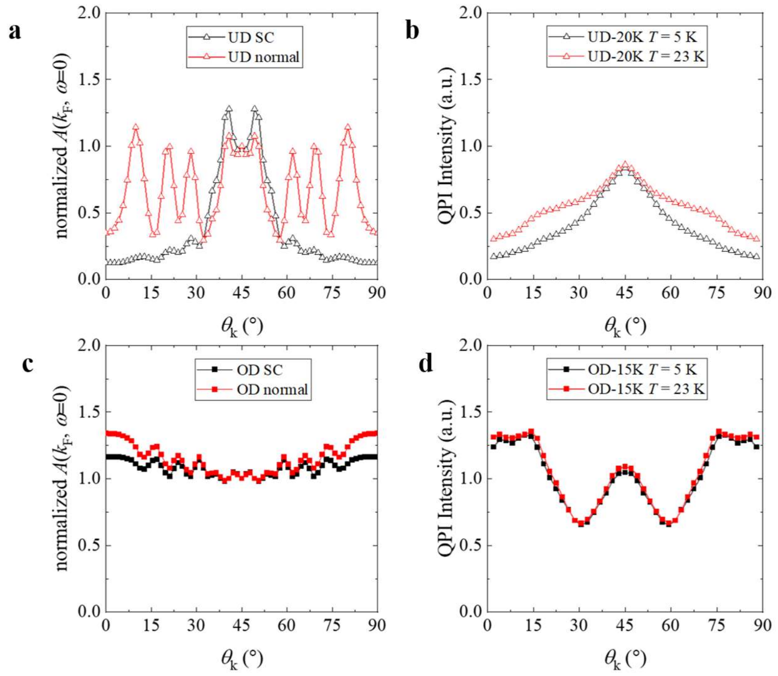

Fig. S12| Comparison between experimental and simulated results. a, The angle dependence of simulated spectral function at E_F in the normal state and superconducting state of the underdoped configuration. The θ_k at 0° (90°) and 45° corresponds to antinodal and nodal region, respectively. **b**, The QPI intensity of the UD-20K sample. **c-d**, The same data set as **a-b** in the overdoped configuration and the OD-15K sample.

XIII. <u>The evaluation of the superconducting gap size</u>

To give an accurate estimation of the superconducting gap in different locations in the OD-15K sample, we use a *d*-wave Dynes formula to fit each spectrum in a dense grid. Considering the existence of vHS, the non-constant normal DOS background would affect the bare Dynes formula. We divide each spectrum at $T = 5$ K by the normal state spectrum on the same position, and then fit it using the *d*-wave Dynes formula with thermal and instrumental broadening in the least square method. The *d*-wave Dynes formula is expressed as:

$$\mathrm{DOS}(E) \propto \int_0^{2\pi} \mathrm{Re}\left\{\frac{E + \mathrm{i}\Gamma}{\sqrt{(E + \mathrm{i}\Gamma)^2 - \Delta_{\mathbf{k}}^2}}\right\} \mathrm{d}\theta_{\mathbf{k}}$$

The Δ_k is taken to the standard *d*-wave form $\Delta_\mathrm{k} = \Delta(\cos k_\mathrm{x} - \cos k_\mathrm{y})/2$, where Δ is the gap size at antinodes. We display the superconducting gap size Δ and quasiparticle scattering rate Γ in Fig. S13 The superconducting gap size is centering at 5.9 meV, which is consistent with the previous report (*Nat. Phys.* 3, 802 (2007)). The gap to T_c ratio is much larger than the weak-coupling *d*-wave BCS ratio $2\Delta = 4.3k_\mathrm{B}T_\mathrm{c}$, confirming that overdoped Bi-2201 is beyond the BCS paradigm. Besides, the quasiparticle broadening evaluates the scattering rate of the quasiparticle $\Gamma \sim \hbar/\tau$, which is centered at 5.5 meV. This scattering rate meets the $\hbar/\tau \sim \Delta_\mathrm{SC}$ condition to generate gapless quasiparticles. To quantitively compare the decoherence of quasiparticle, we also extracted the gap size and the quasiparticle broadening with the polynomial background[8]. As shown in Fig. S13e-h, the superconducting gap size is centered at 9.5 meV and quasiparticle broadening is centered at 2.0 meV, which is sharp contrast with on in OD-15K sample. And it is consistent with the absence of emergent normal fluid in OP-32K, because of non-compliance of condition $\Delta \sim \hbar/\tau$.

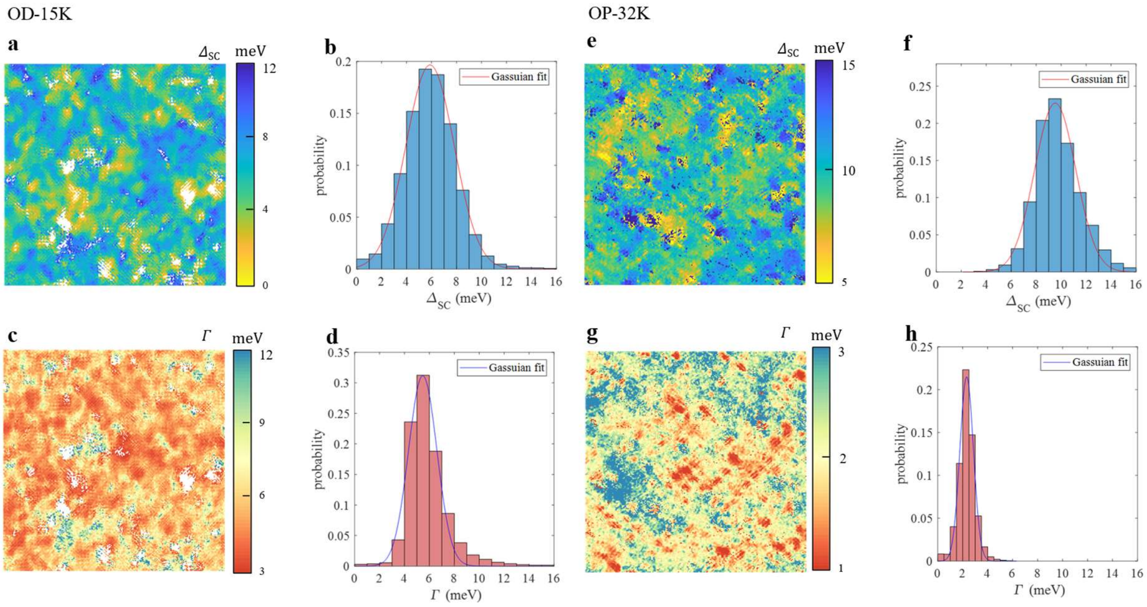

Fig. S13| Distribution of superconducting gap and quasiparticle broadening in OD-15K and OP-32K samples. a,c, The spatial distribution of the gap size Δ and quasiparticle broadening Γ in OD-15K sample, respectively. **b,d,** The histogram of the Δ and Γ, respectively in OD-15K sample. The gaussian peak fitting is also displayed. **e-h,** Same dataset with **a-d** but in OP-32K sample.

XIV. Absence of zero-energy arc-like QPI in bilayer cuprate Bi-2212

To explore the normal carrier QPI in other cuprate system, we have studied overdoped bilayer cuprate Bi-2212 with $T_c = 66$ K and $p \sim 0.22$. The susceptibility characterization and atomically resolved topography are displayed in Figs. S14a-b, respectively. The spatially averaged dI/dV spectrum taken on the area shown in Fig. S14b is displayed in Fig. S14c. The dI/dV curve reveals a superconducting gap ~ 18 meV. The sharp superconducting coherence peaks without pseudogap is distinctively different from that in Bi-2201.

Figures S14d-h display the Fourier transform of conductance map $g(\mathbf{q}, E)$ with bias at -40 mV, -10 mV, 0 mV, 10 mV and 30 mV, respectively. Except for zero bias, they all show the nearly-arc-like antinodal QPI, which is highly similar to the QPI patterns in Fig. 3. But there is a key

difference, namely the nearly-circular QPI is completely absent at zero bias in Bi-2212, indicating that there is no normal carrier at zero energy. There are two likely reasons. First, the bulk Bi-2212 cannot be sufficiently overdoped to reduce the antinodal superconducting gap size to satisfy the condition $\frac{\hbar}{\tau} \sim \Delta_{SC}$. Second, the Fermi level of this overdoped sample is still far from the vHS, thus the antinodal band is not flat enough to have a large DOS for enhanced disorder scattering. The absence of zero-energy QPI in this overdoped Bi-2212 is also consistent with the low temperature electronic specific heat results showing the absence of uncondensed electrons specific heat in Bi-2212 with $p \leq 0.22$[9]. Further increase of doping level in Bi-2212 might need the technique of *in situ* ozone annealing[10]. The combination of ozone annealing and STM still needs further efforts because of the surface sensitivity of STM experiment.

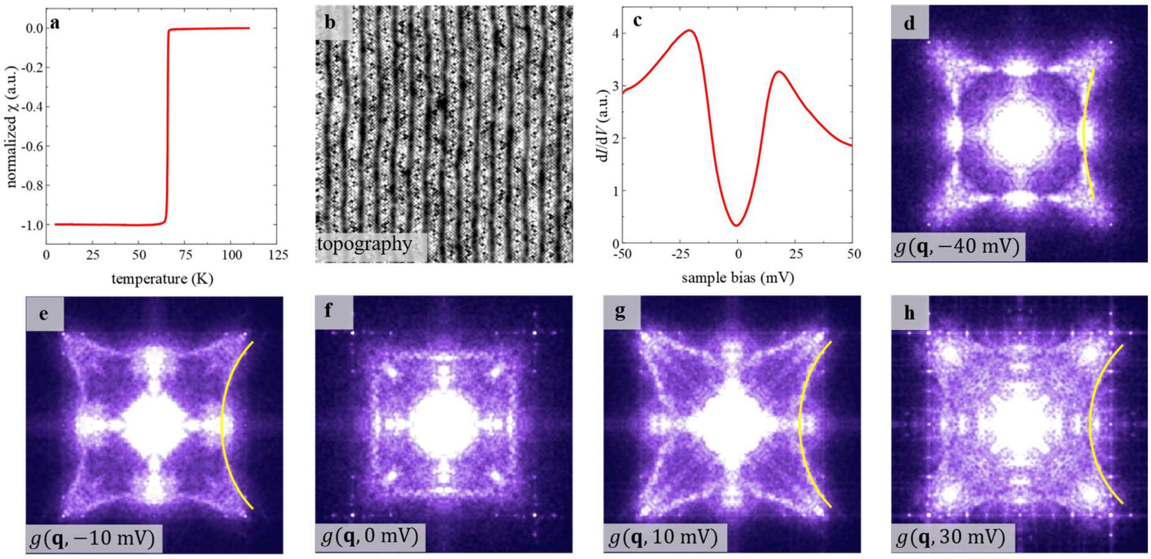

Fig. S14| QPI pattern in an overdoped Bi-2212. a, The temperature dependent susceptibility of an overdoped Bi-2212 with T_c = 66 K. **b**, The topography of the overdoped Bi-2212 with atomically resolved Bi-O surface. **c**, The spatially averaged d*I*/d*V* spectrum taken on the same field of view as **b**. The averaged superconducting gap is ~ 18 meV. **d-h**, The Fourier transform of conductance map *g*(**q**, *E*) with bias at -40 mV, -10 mV, 0 mV, 10 mV and 30 mV, respectively. The arc-like normal carrier QPI is absent at zero bias.

XV. The un-symmetrized QPI dataset

We use the symmetrization process to quantitively extract the intensity of QPI wavevectors, which has been a common practice in similar works. The symmetrization process is executed along the high-symmetry axis of lattice, namely the $q_{x,y}$=0 and q_x±q_y=0 axes. The un-symmetrized patterns of zero-bias QPI are showed in Fig. S15a-g, with the circle indicating the Fermi surface. The un-symmetrized QPI pattern already displays the clear arc-like QPI pattern. Figures S15h-n display the symmetrized images of Fig. S15a-g.

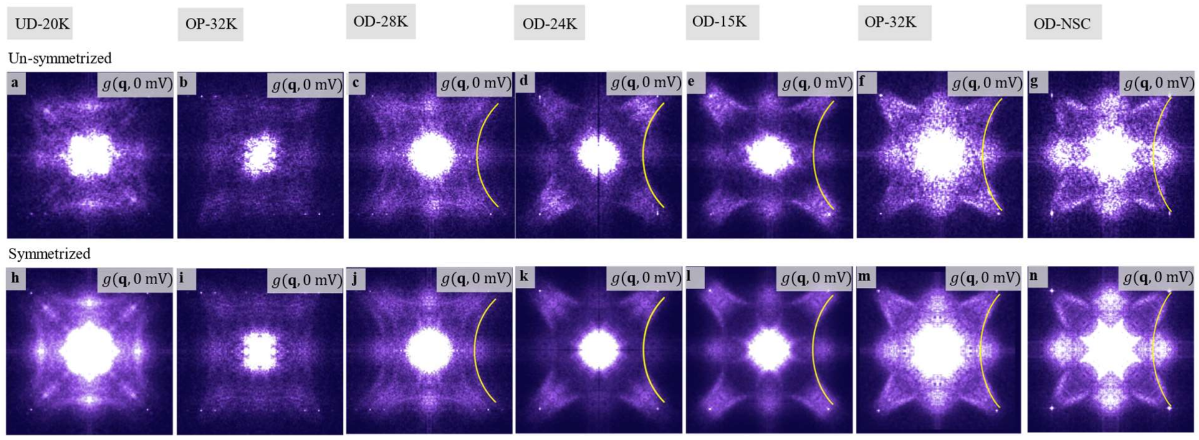

Fig. S15| The un-symmetrized and symmetrized QPI patterns at zero bias. a-g, Un-symmetrized QPI patterns of all samples. The arc-like QPI pattern emerges in overdoped regime. **h-n**, symmetrized QPI patterns.

XVI. The QPI pattern with different bias modulations

In spectroscopic imaging STM experiment, an appropriate bias modulation should be chosen. A larger bias modulation helps enhance the signal-to-noise ratio, but it also broadens the energy resolution. To make sure the results presented in the main text are free of artifact, we repeated the measurements with smaller bias modulations in the same area and tip condition of the

OD-28K sample. The QPI patterns measured with bias modulations 2.1 mV_{rms} and 1 mV_{rms} are displayed in Fig. S16a and S16b, which are nearly identical. It rules out the possibility of artifacts related to bias modulation.

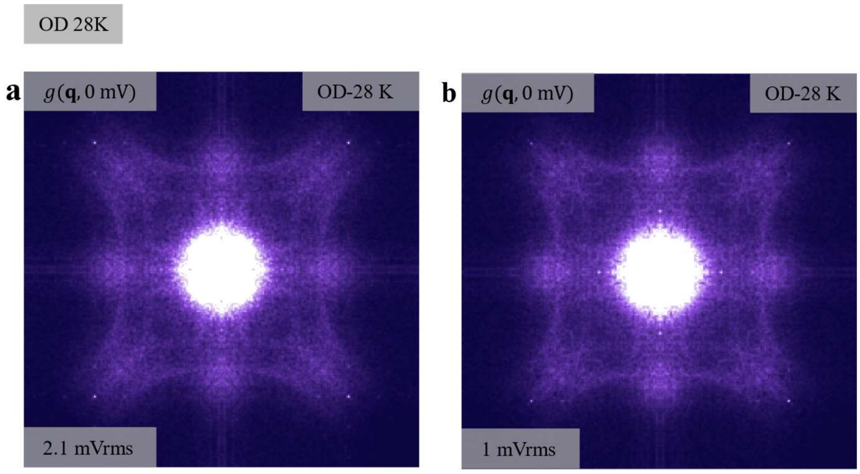

Fig. S16| Arc-like QPI pattern at different bias modulations on the OD-28K sample. a-b, The zero-bias QPI pattern taken by 2.1 and 1 mV_{rms} bias modulations on the OD-28K sample, respectively.

Sample	x (Pb content)	y (La content)
UD-20K	0	0.6
OP-32K	0	0.4
OD-28K	0	0.4
OD-24K	0.38	0
OD-15K	0.38	0
OD-3K	0.24	0
OD-NSC	0.24	0

Tab S1| The details of La and Pb content in every Bi-2201 samples.

Sample	Field of View	Pixel	V_{set}	I_{set}	V_{mod}	Temperature
UD-20K	$42 \times 42\ nm^2$	256×256	-100 mV	120 pA	1.4 mV	5K and 23 K
OP-32K	$41 \times 41\ nm^2$	256×256	-100 mV	200 pA	2.1 mV	5K
OD-28K	$49 \times 49\ nm^2$	285×285	-100 mV	300 pA	2.1 mV	5K
OD-24K	$48 \times 48\ nm^2$	256×256	-150 mV	200 pA	2.1 mV	5K
OD-15K	$42 \times 42\ nm^2$	256×256	-100 mV	250 pA	1.7 mV	5K and 23 K
OD-3K	$30 \times 30\ nm^2$	256×256	-100 mV	300 pA	2.1 mV	5K
OD-NSC	$36 \times 36\ nm^2$	256×256	-100 mV	300 pA	2.1 mV	5K

Tab. S2| The parameters of spectral grid supporting the analysis in the main text.

References

1. Kurashima, K. *et al.* Development of ferromagnetic fluctuations in heavily overdoped (Bi, Pb) $_2Sr_2CuO_{6+\delta}$ copper oxides. *Phys. Rev. Lett.* **121**, 057002 (2018).
2. Ando, Y. *et al.* Carrier concentrations in $Bi_2Sr_{2-z}La_zCuO_{6+\delta}$ single crystals and their relation to the Hall coefficient and thermopower. *Phys. Rev. B* **61**, R14956–R14959 (2000).
3. He, Y. *et al.* Fermi surface and pseudogap evolution in a cuprate superconductor. *Science* **344**, 608–611 (2014).
4. Li, X. *et al.* Quasiparticle interference and charge order in a heavily overdoped non-superconducting cuprate. *New J. Phys.* **20**, 063041 (2018).
5. Lawler, M. J. *et al.* Intra-unit-cell electronic nematicity of the high-T_c copper-oxide pseudogap states. *Nature* **466**, 347–351 (2010).
6. Haas, S., Balatsky, A. V., Sigrist, M. & Rice, T. M. Extended gapless regions in disordered d_{x2-y2} wave superconductors. *Phys. Rev. B* **56**, 5108–5111 (1997).
7. Zou, C. *et al.* Particle–hole asymmetric superconducting coherence peaks in overdoped cuprates. *Nat. Phys.* **18**, 551–557 (2022).
8. Tromp, W. O. *et al.* Puddle formation and persistent gaps across the non-mean-field breakdown of superconductivity in overdoped $(Pb,Bi)_2Sr_2CuO_{6+\delta}$. *Nat. Mater.* **22**, 703–709 (2023).
9. Loram, J. W., Luo, J., Cooper, J. R., Liang, W. Y. & Tallon, J. L. Evidence on the pseudogap and condensate from the electronic specific heat. *J. Phys. Chem. Solids* **62**, 59–64 (2001).
10. Drozdov, I. K. *et al.* Phase diagram of $Bi_2Sr_2CaCu_2O_{8+\delta}$ revisited. *Nat. Commun.* **9**, 5210 (2018).
